# Assessment of genetic diversity in Ethiopian field pea (*Pisum sativum* L.) accessions with newly developed EST-SSR markers

**DOI:** 10.1186/s12863-015-0261-5

**Published:** 2015-08-19

**Authors:** Abel Teshome, Tomas Bryngelsson, Kifle Dagne, Mulatu Geleta

**Affiliations:** Department of Plant Breeding, Swedish University of Agricultural Sciences, Box 101, SE-23053 Alnarp, Sweden; Department of Microbial, Cellular and Molecular Biology, Addis Ababa University, P. O. Box 1176, Addis Ababa, Ethiopia

**Keywords:** Genetic diversity, EST-SSR, Ethiopia, Field peas, *Pisum sativum*, Polymorphism

## Abstract

**Background:**

Field pea (*Pisum sativum* L.) is among the prominent crops in the world as food and feed. There are relatively few simple sequence repeat (SSR) markers developed from expressed sequence tags (ESTs) in *P. sativum.*

**Results:**

In the present study, 15 new EST-SSR markers were developed from publicly available ESTs. These markers have successfully amplified their target loci across seven *Pisum sativum* subsp. *sativum* accessions. Eleven (73 %) of these SSRs were trinucleotide repeats, two (13 %) dinucleotide and two (13 %) were hexanucleotide repeats. Across-taxa transferability of these new markers was also tested on other subspecies of *Pisum* as well as on *P. fulvum*, *Vicia faba* and *Lens culinaris*. In *Pisum sativum* subsp. *sativum*, 13 of the 15 markers were polymorphic and 12 of them subsequently used for genetic diversity analysis. Forty six accessions, of which 43 were from Ethiopia, were subjected to genetic diversity analysis using these newly developed markers. All accessions were represented by 12 individuals except two (NGB103816 and 237508) that were represented by 9 and 11 individuals, respectively. A total of 37 alleles were detected across all accessions. PS10 was the most polymorphic locus with six alleles, and the average number of alleles per locus over the 12 polymorphic loci was 3.1. Several rare and private alleles were also revealed. The most distinct accession (32048) had private alleles at three loci with 100 % frequency.

**Conclusion:**

These newly developed EST-SSR primer-pairs successfully amplified expected loci in *P. sativum* subsp. *sativum* as well as in other subspecies of the genus *Pisum* and related genera. High levels of genetic variation were detected in field pea accessions from Ethiopia using these markers. This result implies the potential of the Ethiopian field pea gene pool for improvement of field peas in various desirable traits. In addition, these markers could be a valuable asset in resolving the inconsistency in the taxonomic status of the different subspecies of genus *Pisum* as well as for characterization of field pea accessions in different gene banks around the world for breeding and conservation purposes.

**Electronic supplementary material:**

The online version of this article (doi:10.1186/s12863-015-0261-5) contains supplementary material, which is available to authorized users.

## Background

Field pea (*Pisum sativum* L.) is one of the ancient and prominent crops. Along with major cereals, like wheat and barley, it signaled the end of the gathering and hunting era by man-kind [1, 2]. Currently, field pea is an important source of food in developing countries and a major feed in the developed world. Ethiopia holds the number one spot in Africa and sixth in the world in field pea production [3]. However, local landraces are becoming low yielding and less profitable to subsistence farmers. The reduction in yield is due to pests like pea weevil (*Bruchus pisorum* L.) and pea aphid (*Acyrthosiphon pisum*); diseases like Ascochyta blight (*Ascochyta pisi*) and powdery mildew (*Erysiphe polygoni*), and climatic changes [4, 5]. To halt further economic loss in the context of unpredictable climatic changes and biotic stresses, there is an urgent need to breed for more resistant and high yielding varieties. Characterization of available gene pools is the primary step for developing high yielding and resilient varieties adapted to local environments. There are more than 2000 pea accessions at the Ethiopian Institute of Biodiversity (EIB) which constitute a valuable resource in field pea breeding. However, only few of these accessions have been properly characterized for their genetic diversity and/or agronomic traits [4–6].

For characterization of large number of landraces in a relatively short time at low costs, the use of simple sequence repeat (SSR) markers is an ideal approach. SSR markers’ even distribution throughout the genome, co-dominant inheritance, multi-allelic nature, easy detection by PCR and high reproducibility make them convenient for whole genome characterization [7–9]. SSR markers have been used to assess genetic diversity in field peas and were found to be very effective [10, 11]. Developing Expressed Sequence Tag based SSRs (EST-SSRs) from available EST sequences in the public databases is relatively simple and straight forward. Furthermore, EST-SSRs have higher rate of transferability across related species and genera than anonymous genomic SSRs [12, 13], and hence are preferable for some applications such as phylogenetic studies and QTL mapping. Since there are relatively few EST-SSR markers developed in *P. sativum* in comparison with cereal crops, the study of its transcribed part of the genome remains important until the whole genome gets sequenced. In the present study, new EST-SSR markers were developed from publicly available *P. sativum* ESTs. These microsatellites were then tested for transferability to related genera and for genetic diversity analysis of *P. sativum* ssp. *sativum*.

## Methods

### Development of EST-SSR markers

A total of 9377 pea EST sequences from National Center for Biotechnology Information (NCBI) were analyzed for mining SSRs with two to six repeat motifs using Msatcommander-0.8.1 (http://code.google.com/p/msatcommander/) [14]. The analysis revealed that 7 % (646) of these sequences contain SSRs. After excluding similar, over-lapping and very short sequences as well as sequences with more than one SSRs, 100 sequences containing SSRs were chosen as candidates for designing primers using the Primer3 primer designing program [15]. SSR primer-pairs were successfully designed for 37 of the 100 sequences. Fifteen of these primer-pairs consistently amplified their targets in 84 individual samples representing seven *P. sativum* subsp. *sativum* accessions (Additional file 1).

### Plant material

Seven accessions of *P. sativum* L. subsp. *sativum* from Ethiopia were used for primer development (Additional file 1). These accessions were chosen to represent the four major field pea producing regional states in Ethiopia. Across-taxa transferability of the microsatellite was tested on other *Pisum* species and subspecies as well as on two other legumes, *Vicia faba* L. and *Lens culnaris* L. (Additional file 2). All accessions used in across-taxa transferability analysis of the markers were obtained from NordGen.

Among the newly developed EST-SSRs, 12 polymorphic loci were used for genetic diversity analysis of 46 *P. sativum* subsp. *sativum* accessions (Additional file 1). All accessions were represented by 12 individuals except two (NGB103816 and 237508) that were represented by 9 and 11 individuals, respectively. Forty three of the 46 accessions were landraces obtained from Ethiopia (EIB collections) (Additional file 1). The selection of these landraces was based on site of collection and altitude. The remaining three accessions were varieties from NordGen (Additional file 3). These varieties were included to compare the level of genetic differentiation between Ethiopian accessions and accessions from other countries.

### DNA isolation and PCR amplification

Extraction of DNA and quality control procedures were performed as reported in Geleta et al. [16]. A total volume of 24 μl containing 2.5 μl PCR buffer (10 mM Tris–HCl pH 8.3, 50 mM KCl), 0.3 mM of each dNTPs, 0.3 mM each of forward and reverse primers, 1 U (0.04 U/μl) Dream Tag polymerase (Sigma, Germany) and 15 ng of DNA was used for PCR reaction. Negative controls were included with sterile millipore water replacing DNA, as a quality control measure. A 50 bp DNA ladder (GeneRuler™, Fermentas Life Sciences) was used as reference when the PCR products were electrophoresed using 1.2 % agarose gels.

The PCR amplification was performed in 96-well plates using the GeneAMP PCR system 9700 thermo cycler (Applied Biosystems Inc. USA) with the following temperature profiles: initial denaturation at 95 *°*C for 3 min followed by nine touchdown cycles of denaturation at 94 *°*C for 30 s, 30 s annealing at 61 *°*C reducing by −1 *°*C every cycle and 45 s extension at 72 *°*C. Afterwards, 29 cycles of denaturation at 94 *°*C for 30 s, annealing at 51 *°*C for 30 s and extension at 72 *°*C for 45 s were carried out with a 20 min final extension step at 72 *°*C. The amplified products were kept at 4 *°*C until electrophoresis.

The forward primers were 5′-labeled with 6-FAM™ or HEX™ fluorescent dyes. In order to prevent non-template addition by *Taq* polymerase to the PCR products, the reverse primers were PIG-tailed with *GCTTCT* as reported in [17]. Multiplexing of the PCR products into panels was done as described in Geleta et al. [16]. The capillary gel electrophoresis of the PCR products was done using an ABI Prism 3730 DNA Analyzer (Applied Biosystems) at the Department of Plant and Environmental Sciences, University of Copenhagen, Denmark.

### Data analysis

GeneMarker® V2.2.0 software (SoftGenetics, LLS, State College, Pennsylvania) was used for peak identification and fragment sizing. The size of the PCR products was determined based on the Genescan-500 LIZ internal size standard. In all cases, default settings in Genemarker were applied for detection of bands with the recommended threshold intensity of 200 but peaks were accepted for scoring after they were manually checked.

POPGENE software version 1.31 was used to calculate genetic diversity parameters for each locus [18]. Arlequin 3.0 was used for analysis of molecular variance (AMOVA) according to Excoffier et al. [19]. Free-Tree Freeware program [20] was used to generate genetic distance coefficients, and for cluster analysis and bootstrapping. Trees generated by Free-Tree were viewed using TreeView (Win32) 1.6.6 program [21]. STRUCTURE software [22] was used for analysis of population structure based on data generated from 12 SSR loci. The admixure model with 10,000 burning periods and 100,000 replicates was used to estimate K value, with ten independent runs (K = 1 to 10). Population number was estimated as described by [23] using the STRUCTURE HARVESTER software [24]. CLUMPP software [25] was used to align the clusters across the replicates whereas the population clusters were depicted using the DISTRUCT software [26].

## Results

The list of 15 new EST-SSR loci developed in this study is given in Table 1 along with their source sequence accession numbers, primer-pairs, repeat motifs and fragment size range. All primer-pairs were successful in amplifying target loci in all accessions of *P. sativum* subsp. *sativum*. Eleven of the 15 SSR loci have trinucleotide repeats, two are dinucleotide repeats (PS20 and PS21) and two are hexanucleotide repeats (PS05 and PS11). Two of the 15 loci (PS09 and PS20) were monomorphic whereas the remaining 13 loci were polymorphic across the seven *P. sativum* subsp. *sativum* accessions initially tested (Table 2). The across-taxa transferability analysis of these loci revealed that all 15 loci were amplified in all seven taxa of *P. sativum* (Table 2). Most of these loci were monomorphic in *P. sativum* subsp. *elatius* var. *pumilio* and *P. sativum* subsp. *transcaucasicum* (Table 2). On the other hand, 12 of the 15 loci were polymorphic in *P. sativum* subsp. *asiaticum* across the three individual plants used. All loci except PS04 were also successfully amplified in *P. fulvum*, but all of them were monomorphic among the four individual plants representing this species. Interestingly, nine of the 15 loci were amplified in *V. faba*, of which three (PS05, PS08 and PS16) were polymorphic. In the case of *L. culinaris*, 10 of the 15 loci were amplified, all of which were monomorphic among the two individuals tested.

**Table 1:** Primer-pairs, repeat motif and allele size range of newly developed EST-SSR loci

Locus name	SSAN^b^	forward primer (5′- 3′)	reverse primer (3′- 5′)	Expected sizes	Repeat motif	Allele size range
PS01	cl191ct198cn251e00	TTCGTTTTGGTTACGATCGAG	AGGTGTGGTTCAGAGGCTGT	247	(ATT)_7_	244-253
PS04	cl41ct42cn53e00	CAGCACTATCAATCACCACACTG	GCGTTGTTCCTGTTTCACCT	292	(AAT)_14_	265-298
PS05^a^	PSS08K23u	TGCATGCAGGACACTTGTAG	AAAAGTGCGTGGACCTGAAC	291	(AGAGTT)_5_	261-297
PS08	PSC22C12r	TCACAGGAGGAAAAGGATGG	GGAGGGGGTTTGGTAAAAAG	189	(CCT)_6_	168-198
PS09^a^	PSC23H12u	TCAAGGGGAAACTGGAAAAA	TCATGATGTGGAGGCCAAG	220	(GGT)_6_	223-229
PS10	PSC24E20u	TCCATTCTCCAACAACACTAACA	TGGCTGAACTCACCAAACAA	396	(ATC)_7_	405-450
PS11	PSC25H08u	TGGGAAACTTGAGCCTACGA	TGAAACAGCCTCTTCTTCAATTA	299	(GGCGGT)_4_	299-311
PS13	PSC28N15u	TCCTACCTACCTACCCTTTCCA	TGTGGACCCCACCATAAGAC	234	(ATC)_7_	225-261
PS15	PSC30D03r	AGAATGGCGAGACAACGAAG	AACAACAGGTGCCAAGGAAG	204	(GGT)_7_	210-213
PS16	PSC32L05u	TTTCTGCTTCTATTGCATGTTACC	TCCATTTGCTACACCAATATTTTCT	250	(ATT)_7_	256-286
PS18	PSC33A19r	AGGATGGCAAGACAGAGAAGA	TGGAGGAATGGGAGGTAGTG	156	(CCT)_6_	156-165
PS20^a^	PSC35B08u	GGCTCCAACATGAAACAACA	TGGCTGATCCTGTCAACAAC	217	(AT)_6_	211-225
PS21	PSC35D07u	AAGTGTACAAAATTACAATGGCACA	GTGGACCTCGTCGTTCAAGT	393	(AT)_19_	361-405
PS34	PSS19H07u	CCGAAACAAGTTCGACAAAAA	TGTTGATGAAGCTGCCAATG	218	(CTT)_6_	221-224
PS36	PSS21P12u	ATGGCCCAATATTCAAACGA	GCTCAAGCTGCAATAACAACC	248	(GTT)_6_	209-218

The allele size range refers to allele size across all taxa included in the study

^a^The loci were not used for genetic diversity analysis. ^b^SSAN = Source sequence accession number

**Table 2:** Transferability and polymorphism of SSR loci among different taxa of the genus *Pisum* and related genera

Loci	Taxa/number of samples analyzed									
	sativum^a^/84	abyssinicum^b^/6	asiaticum^c^/3	elatius^d^/6	pumilio^e^/4	Trans-caucasicum^f^/3	arvense^g^/6	fulvum^h^/4	faba^i^/6	culnaris^j^/2
PS01	p	p	p	p	m	m	m	m	m	m
PS04	p	p	p	p	m	m	p	-	m	-
PS05	p	m	p	p	m	m	m	m	p	-
PS08	p	m	p	p	m	m	p	m	p	m
PS09	m	p	p	m	m	m	m	m	-	m
PS10	p	p	p	m	m	p	p	m	-	-
PS11	p	p	p	m	m	m	m	m	m	m
PS13	p	p	p	p	m	m	p	m	-	-
PS15	p	p	p	p	p	m	p	m	m	m
PS16	p	m	m	p	m	m	p	m	p	m
PS18	p	p	p	p	m	m	m	m	m	m
PS20	m	p	p	p	m	m	m	m	m	m
PS21	p	p	p	p	m	p	p	m	-	m
PS34	p	p	m	p	m	m	m	m	-	-
PS36	p	m	m	m	m	m	m	m	-	m

^a^ *P. sativum* subsp. *sativum*; ^b^ *P. sativum* subsp. *abyssinicum*; ^c^ *P. sativum* subsp. *asiaticum*; ^d^ *P. sativum* var. *elatius*; ^e^ *P. sativum* subsp. *elatius* var *Pumilio*; ^f^ *P. sativum* subsp. *transcaucasicum*; ^g^ *P. sativum* var. arvense; ^h^ *P. fulvum*; ^i^ *V. faba* L; ^j^ *L. culinaris*

The numbers after the code for each taxa refer to the number of individuals representing each taxon

p: locus amplified and polymorphic

m: locus amplified but monomorphic within taxon

-: locus not amplified

In Total, 37 alleles were detected across the 12 loci of the 46 accessions genotyped. The observed number of alleles (na) per locus ranged from two (in PS08, PS15, PS18, PS34 and PS36) to six (in PS10; Table 3). The effective number of alleles (ne) at each locus varied from 1.05 (PS08 and PS34) to 2.83 (PS16). Observed heterozygosity (Ho) ranged from zero at loci PS04, PS08, PS18, PS21, PS34 and PS36 to 0.05 at locus PS13 (Table 3). The Shannon diversity index (I) per locus ranged from 0.11 for PS08 and PS34 to 1.13 for PS16. The average Shannon diversity index for all loci was 0.53.

**Table 3:** Genetic diversity estimates of microsatellite loci based on 46 *P. sativum* subsp. *sativum* accessions

Locus	Sample Size	na	ne	I	Ho	He	Av. He	Fst
PS01	1044	3	2.07	0.88	0.01	0.52	0.29	0.44
PS04	1000	5	1.28	0.46	0.00	0.22	0.14	0.45
PS08	1088	2	1.05	0.11	0.00	0.04	0.00	1.00
PS10	1084	6	2.10	0.96	0.03	0.52	0.33	0.37
PS11	1074	3	1.62	0.67	0.03	0.38	0.23	0.39
PS13	1050	3	2.01	0.86	0.05	0.50	0.34	0.35
PS15	954	2	1.92	0.67	0.04	0.48	0.33	0.40
PS16	1088	4	2.83	1.13	0.03	0.65	0.42	0.35
ps18	1088	2	1.06	0.14	0.00	0.06	0.01	0.82
PS21	898	3	1.08	0.18	0.00	0.08	0.02	0.80
PS34	1086	2	1.05	0.11	0.00	0.04	0.00	1.00
PS36	1070	2	1.06	0.13	0.00	0.05	0.01	0.85
Mean	1044	3.08	1.59	0.53	0.02	0.30	0.18	0.60
St. Dev		1.31	0.59	0.38	0.02	0.24	0.16	

*na* observed number of alleles, *ne* effective number of alleles, *I* Shannon information index, *Ho* observed heterozygosity, *He* expected heterozygosity, *Av. He* average heterozygosity, *Fst* F statistics

**Fig. 1:**
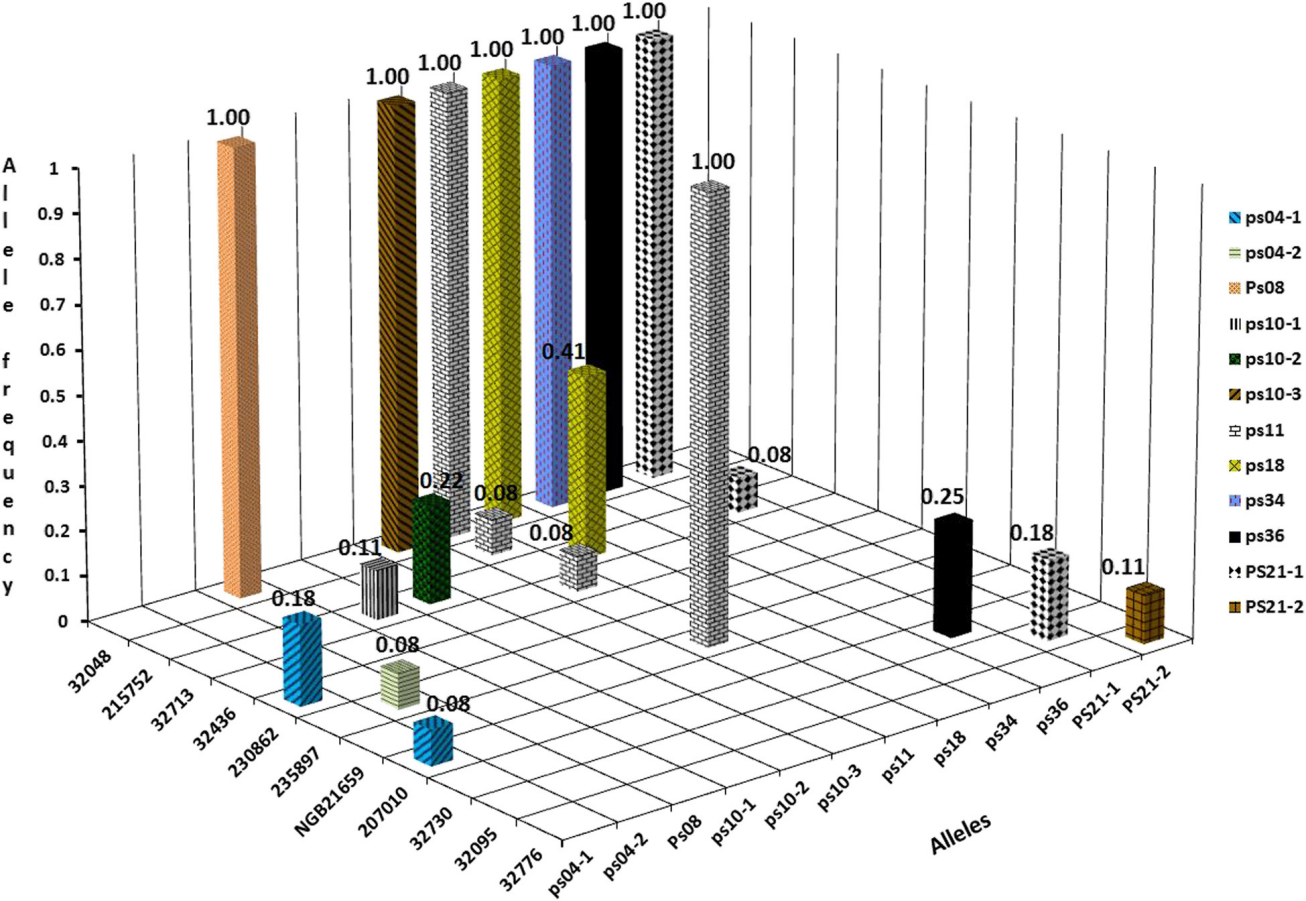
Accessions of *P. sativum* subsp. *sativum* with rare or private alleles or with alleles limited to few accessions at the EST-SSR loci studied. The frequency of each allele for each accession is given on top of each bar

It is interesting to note that only five of the 46 accessions were required to capture all the 37 alleles, namely: 207010, 235897, 32048, 32713 and 32776. The average frequency of each allele across these accessions was more than 1 % (Table 4). Loci like PS04, PS10 and PS21 produced rare allele(s) restricted to one to two accessions with frequencies of less than 20 % and have overall frequencies of less than 1 % across the 46 accessions. Private alleles unique to a single accession were also amplified with 100 % frequency at loci PS08, PS10, and PS34 (Fig. 1). Alleles restricted to 2, 2, 3 and 4 accessions were amplified at loci PS18, PS36, PS21 and PS11, respectively, at frequencies ranging from 8 % to 100 % (Fig. 1). Furthermore, PS15 and PS21 scored null alleles in all individuals of an accession from Norway (NGB21659).

**Table 4:** List of the smallest number of accessions that jointly produced all the 37 alleles across the 12 EST-SSR loci and their allele frequencies

Alleles	Allele frequency per accession					MAF^a^	Alleles	Allele frequency per accession					MAF^a^
	207010	235897	32048	32713	32776			207010	235897	32048	32713	32776	
PS01-1	0.08	0.00	0.00	0.00	0.00	0.02	PS13-3	0.33	0.13	0.00	0.08	0.17	0.14
PS01-2	0.25	0.08	1.00	0.50	0.08	0.38	PS15-1	0.82	0.79	0.00	0.71	0.40	0.54
PS01-3	0.67	0.92	0.00	0.50	0.92	0.60	PS15-2	0.18	0.21	1.00	0.29	0.60	0.46
PS04-1	0.00	0.33	1.00	0.10	0.00	0.29	PS16-1	0.50	0.21	0.00	0.29	0.50	0.30
PS04-2	0.08	0.00	0.00	0.00	0.00	0.02	PS16-2	0.00	0.17	0.00	0.46	0.00	0.13
PS04-3	0.00	0.00	0.00	0.00	0.17	0.03	PS16-3	0.50	0.63	0.58	0.25	0.50	0.49
PS04-4	0.92	0.58	0.00	0.90	0.83	0.65	PS16-4	0.00	0.00	0.42	0.00	0.00	0.08
PS04-5	0.00	0.08	0.00	0.00	0.00	0.02	PS17-1	0.50	0.21	0.00	0.29	0.50	0.30
PS08-1	0.00	0.00	1.00	0.00	0.00	0.20	PS17-2	0.00	0.17	0.00	0.46	0.00	0.13
PS08-2	1.00	1.00	0.00	1.00	1.00	0.80	PS17-3	0.50	0.63	0.58	0.25	0.50	0.49
PS10-1	0.08	0.00	0.00	0.06	0.17	0.06	PS17-4	0.00	0.00	0.42	0.00	0.00	0.08
PS10-2	0.58	0.88	0.00	0.61	0.67	0.55	ps18-1	0.00	0.00	1.00	0.42	0.00	0.28
PS10-3	0.33	0.13	0.00	0.00	0.17	0.13	ps18-2	1.00	1.00	0.00	0.58	1.00	0.72
PS10-4	0.00	0.00	0.00	0.11	0.00	0.02	PS21-1	0.00	0.00	1.00	0.08	0.00	0.22
PS10-5	0.00	0.00	0.00	0.22	0.00	0.04	PS21-2	1.00	1.00	0.00	0.92	0.89	0.76
PS10-6	0.00	0.00	1.00	0.00	0.00	0.20	PS21-3	0.00	0.00	0.00	0.00	0.11	0.02
PS11-1	0.17	0.13	0.00	0.54	0.58	0.28	PS34-1	0.00	0.00	1.00	0.00	0.00	0.20
PS11-2	0.83	0.88	0.00	0.46	0.42	0.52	PS34-2	1.00	1.00	0.00	1.00	1.00	0.80
PS11-3	0.00	0.00	1.00	0.00	0.00	0.20	PS36-1	1.00	1.00	0.00	1.00	1.00	0.80
PS13-1	0.08	0.00	0.00	0.04	0.17	0.06	PS36-2	0.00	0.00	1.00	0.00	0.00	0.20
PS13-2	0.58	0.88	0.00	0.88	0.67	0.60							

The accessions were represented by 12 individuals

^a^ *MAF* Mean allele frequency

Percentage of polymorphic loci, Shannon diversity index, observed and expected heterozygosity, and mean Nei’s minimum genetic distance were calculated for each accession. The highest percentage of polymorphic loci was 75 %, which was recorded in accession 32713 from the SNNP region (altitude of 2600 a.s.l.), while the least was zero for two accessions from Norway (accessions NGB21659 and NGB7131; Table 5). Apart from two accessions from Ethiopia (230048; 32048), one accession from USA (NGB103816) and the two Norwegian accessions, the percentage of polymorphic loci in each accession was above 40 %. When the percentage of polymorphic loci was compared for each region, the highest value was recorded for the Amhara region (53 %) and the lowest for Tigray (42 %). Correspondingly, the highest Shannon diversity index was recorded for the Amhara region (0.33) and lowest for Tigray with a value of 0.24 (Table 5).

**Table 5:** Percentage of polymorphic loci (%PL), Shannon’s diversity index (I), observed heterozygosity (Ho), expected heterozygosity (He) and mean Nei’s minimum genetic distance (GD) for each accession, estimated based on 12 EST-SSR loci. The corresponding average percent seed damage (PSD) of the accessions by pea weevil (*Bruchus pisorum* L.) during field trials conducted at three sites in Ethiopia during June-October 2011 (Teshome et al. 2014) was given for comparison purpose

Acc^a^	Region/Zone^b^	%PL	I	Ho	He	GD	PSD^c^	Acc^a^	Region/Zone^b^	%PL	I	Ho	He	GD	PSD
32463	Amhara-MG	50.0	0.25	0.00	0.17	0.09	39		**Oromia** ^**m**^	**48.7**	**0.29**	**0.01**	**0.19**	**0.14**	**44**
229824	Amhara-MG	50.0	0.28	0.02	0.18	0.09	41	213964	SNNP-BM	58.3	0.34	0.01	0.23	0.09	36
32466	Amhara_MG	50.0	0.34	0.07	0.25	0.08	31	228068	SNNP-H	41.7	0.37	0.03	0.22	0.07	37
32377	Amhara-SG	50.0	0.25	0.08	0.18	0.08	39	32509	SNNP-G	41.7	0.20	0.03	0.13	0.10	27
235897	Amhara-SG	58.3	0.31	0.03	0.19	0.09	49	236900	SNNP-K	41.7	0.20	0.01	0.12	0.16	41
207010	Amhara-SG	58.3	0.37	0.00	0.24	0.08	44	244799	SNNP-K	50.0	0.26	0.00	0.17	0.08	41
32436	Amhara-SG	50.0	0.4	0.01	0.25	0.07	45	212983	SNNP_Ge	58.3	0.33	0.01	0.22	0.10	39
32789	Amhara-DW	50.0	0.36	0.06	0.26	0.11	44	244801	SNNP-S	41.7	0.21	0.02	0.12	0.15	54
32809	Amhara-DW	58.3	0.34	0.01	0.24	0.09	47	32712	SNNP-SO	50.0	0.32	0.01	0.22	0.09	54
215752	Amhara-O	58.3	0.46	0.01	0.3	0.08	42	225816	SNNP-SO	58.3	0.40	0.03	0.27	0.07	40
229219	Amhara-SS	50.0	0.29	0.02	0.19	0.08	46	32717	SNNP-SO	50.0	0.26	0.00	0.17	0.10	54
	**Amhara** ^**m**^	**53.0**	**0.33**	**0.03**	**0.22**	**0.11**	**42**	32713	SNNP-SO	75.0	0.49	0.05	0.32	0.10	49
230259	B-G	41.7	0.14	0.01	0.08	0.11	38	32715	SNNP-SO	58.3	0.39	0.00	0.25	0.07	42
32006	Oromia_A	50.0	0.29	0.00	0.19	0.08	50	32711	SNNP-SO	41.7	0.28	0.00	0.18	0.12	68
32063	Oromia-A	58.3	0.41	0.00	0.28	0.09	46	32095	SNNP-SO	41.7	0.21	0.02	0.13	0.10	55
230048	Oromia-B	25.0	0.13	0.01	0.08	0.21	66		**SNNP** ^**m**^	**50.6**	**0.30**	**0.02**	**0.19**	**0.12**	**46**
212865	Oromia-B	50.0	0.29	0.01	0.19	0.11	50	237508	Tigray-D	41.7	0.29	0.03	0.19	0.10	63
237950	Oromia-B	50.0	0.25	0.01	0.17	0.14	47	234055	Tigray-M	41.7	0.19	0.01	0.12	0.14	55
230864	Oromia-MrH	41.7	0.21	0.02	0.13	0.20	48		**Tigray** ^**m**^	**41.7**	**0.24**	**0.02**	**0.16**	**0.12**	**59**
230858	Oromia-MsH	41.7	0.2	0.00	0.13	0.18	38	32048	Ethiopia-U	8.3	0.06	0.00	0.05	0.76	46
230862	Oromia-MsH	58.3	0.34	0.03	0.22	0.12	48	32776	Ethiopia-U	66.7	0.41	0.00	0.27	0.09	53
32730	Oromia-J	58.3	0.34	0.02	0.22	0.09	29	NGB21659	Norway-1	0.0	0.00	0.00	0.00	0.26	-
213195	Oromia-J	50.0	0.36	0.01	0.24	0.08	35	NGB7131	Norway-2	0.0	0.00	0.00	0.00	0.13	-
237945	Oromia-MW	41.7	0.23	0.02	0.16	0.09	27		**Norway** ^**m**^	**0.0**	**0.00**	**0.00**	**0.00**	**0.19**	**-**
202282	Oromia-MW	58.3	0.38	0.02	0.24	0.08	41	NGB103816	USA	16.7	0.08	0.01	0.06	0.15	-
203094	Oromia-MS	50.0	0.42	0.00	0.28	0.09	47								

Mean Nei’s minimum genetic distance is a measure of the average genetic distance of each accession from the other accessions. The range of Nei’s minimum genetic distance between accessions was 0002 (accessions 230858 vs 230048) to 0938 (accessions 32048 vs NGB7131)

All accessions were represented by 12 individuals except NGB103816 and 237508, which were represented by 9 and 11 individuals, respectively

^a^ *Acc* accession; ^b^ *SNNP* South nations and nationalities region; *B-G* Benishangul and Gumuz; *MG* Misrak Gojam; *SG* Semen Gonder; *SW* Semen Wello; *DW* Debub Wello; *O* Oromia; *SS* Semen Shewa; *A* Arsi; *B* Bale; *MrH* M’irab Harerge; *MsH* Misrak Harerge; *J* Jimma; *MW* Misrak Welega; *MS* Misrak Shewa; *BM* Bench Maji; *H* Hadiya; *G* Gurage; *K* Kembata; *Ge* Gedeo; *S* Sidama; *SO* Semen Omo; *D* Debubawi; *M* Mehakelawi; *U* Unknown; ^*m*^ mean

The bold data is to give emphasis to the mean values

The mean Nei’s minimum genetic distance of an accession from all other accessions had a range of 0.07 (accessions 32436, 228068, 225816 and 32715) to 0.76 (accession 32048). Other accessions with a relatively high mean genetic distance from the rest of the accessions include NGB21659 (0.26), 230048 (0.21) and 230864 (0.20; Table 5). Interestingly, the mean genetic distance between accessions within regions in Ethiopia was lower than the mean genetic distance between the whole accessions studied, except in the case of Tigray, which was represented by only two accessions. The mean genetic distance between accessions within Amhara region was 0.05 whereas the mean genetic distance between accessions from Amhara and accessions from other origin was 0.11 (Table 6). Similarly, the mean genetic distance between accessions within Oromia region was 0.09 whereas the mean genetic distance between accessions from Oromia and other accessions was 0.14. Excluding accession 32048 (an outlier), the overall mean genetic distance between Ethiopian accessions was 0.08 whereas the mean genetic distance between Ethiopian accessions and accessions from other countries was 0.16 (Table 6).

**Table 6:** Mean Nei’s minimum genetic distance between groups of accessions grouped based on regions within Ethiopia or country of origin

	Amhara^a^	B-G^a^	Oromia^a^	SNNP^a^	Tigray^a^	Ethiopia^b^	Norway	USA	Rest^c^
Amhara	**0.05**	0.04	0.02	0.01	0.02	na	0.05	0.09	0.11
B-G	0.04	na	0.08	0.06	0.04	na	0.08	0.09	0.11
Oromia	0.02	0.08	**0.09**	0.03	0.03	na	0.10	0.12	0.14
SNNP	0.01	0.06	0.03	**0.07**	0.04	na	0.07	0.08	0.12
Tigray	0.02	0.04	0.03	0.04	**0.15**	na	0.07	0.11	0.12
Ethiopia	na	na	na	na	na	**0.08**	0.18	0.13	0.16
Norway	0.05	0.08	0.10	0.07	0.07	0.18	**0.29**	0.16	0.19
USA	0.09	0.09	0.12	0.08	0.11	0.13	0.16	na	0.15

Values in bold refer to mean genetic distance between accessions within a region (country)

*na* not applicable

^a^Different regions within Ethiopia; ^b^Accession 32048 (an outlier) was excluded from the analysis ^c^Values in this column refer to mean genetic distance between groups of accessions from a region (country) given in the corresponding row and the rest of the accessions

**Fig. 2:**
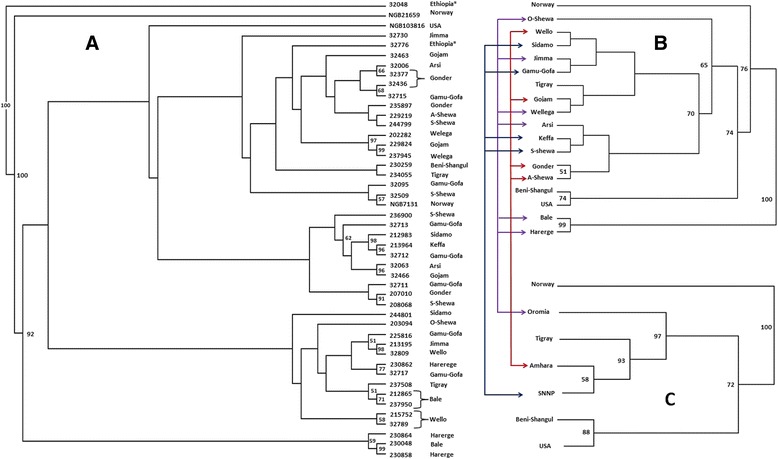
Rogers genetic distance-based UPGMA phenograms for (**a**) 46 accessions of *P. sativum* subsp. *sativum* (sub-region or country of origin of each accession is given in front of the accession name); (**b**) 18 groups of accessions after grouping Ethiopian accessions into sub-region of origin; (**c**) seven groups of the accessions after grouping Ethiopian accessions into their regions of origin. Sub-regions in “B” that belong to the same region are connected to each other and to their corresponding region in “C” with arrows. The two Ethiopian accessions with unknown site of collections (32048 and 32776) were excluded in the case of “B” and “C”. Numbers in front of the branches are bootstrap values (only bootstrap values more than 50 % are shown)

Cluster analysis based on Rogers genetic distance coefficient was done for accessions, sub-regions and regions (Fig. 2). First, accession 32048 was separated from the rest of the accessions with 100 % bootstrap support, and was followed by accession NGB21659 from Norway, also with 100 % bootstrap support. Three accessions from southeastern and eastern Ethiopia (230864, 230048 and 2330858) formed a cluster with 92 % bootstrap support. The other major clusters in Fig. 2a were not supported by high bootstrap values. Accessions NGB103816 (USA) and NGB7131 (Norway) were not separated from Ethiopian accessions. At sub-region level Bale and Harerge formed a separate cluster with 100 % bootstrap support (Fig. 2b). At region level, Amhara region clustered with SNNP with 58 % bootstrap support (Fig. 2c).

Analysis of molecular variance (AMOVA) has revealed significant variation among accessions (P < 0.001) (Table 7). The genetic differentiation among accessions was 41 % while the variation within was 59 %. Furthermore, accessions were pooled into different groups to determine the levels of genetic differentiations among them. In all cases, the genetic differentiation among groups was highly significant even though the levels of differentiations were different. The genetic differentiation between a group of Ethiopian accessions and a group comprising accessions from abroad was 10 % (Table 7). Similarly, only 8 % of the total genetic variation differentiates groups of accessions that were grouped according to regions of origin within Ethiopia. Analysis to determine the level of differentiation among altitudinal groups also gave low in-between variation (2 %).

**Table 7:** Variation among and within all accessions of field peas as well as among and within groups of accessions, grouped based on different criteria

Number of accessions	Source of variation	d.f.	Sum of squares	Variance components	%age of variation	FST	probability
46	Among all accessions	45	521.5	0.47 Va	40.74	0.40	Va and FST = 0.0000
	Within all accessions	1048	699.1	0.68 Vb	59.26		
	Total	1093	1220.6	1.15			
46	Among countries of origin^a^	1	23.3	0.19 Va	10.03	0.10	Va and FST = 0.0000
	Within countries of origin^a^	1092	1835.1	1.76 Vb	89.96		
	Total	1093	1858.4	1.95			
41	Among regions^b^	4	91.7	0.13 Va	7.64	0.08	Va and FST = 0.0000
	Within regions^b^	977	1448.3	1.54 Vb	92.36		
	Total	981	1539.9	1.6			
35	Among altitude groups^c^	2	23.0	0.04 Va	2.11	0.02	Va and FST = 0.0000
	With altitude groups^c^	835	1476.3	1.85 Vb	97.89		
	Total	837	1211.6	1.89			

Different numbers of accessions were included in different groupings because not all accessions fulfil the criteria used for grouping the accessions except in the first and second cases. ^a^ = accessions were grouped into two groups (Ethiopian accessions and accessions from other countries); ^b^ = Accessions from Ethiopia were grouped into five groups according to their regions of origin (Amhara, Beni-Shangul, Oromia, SNNP and Tigray); ^c^ = Accessions from Ethiopia were grouped into three groups based on altitude of collections (<2000; between 2000 and 2500 and > 2500 m asl)

**Fig. 3:**
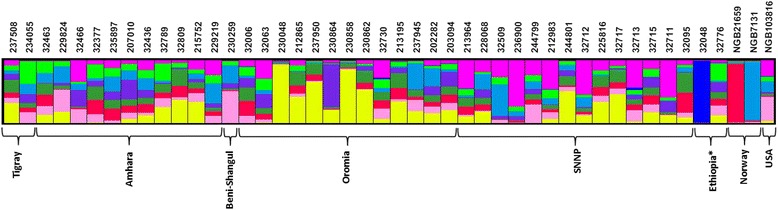
STRUCTURE analysis at *K* = 9 for 46 *P. sativum* accessions. The colors represent different clusters and the colors in each accession represent the average proportion of alleles that placed each accession under two or more clusters. The text above the figure refers to accession names; and the text below the figure refers to different regions within Ethiopia or countries from where the accessions originated. Note: Ethiopia* refers to Ethiopian accessions with unknown site of collection

Based on the 37 alleles distributed over 12 EST-SSR loci, STRUCTURE software was used to analyze population structure. The structure simulation with STRUCTURE HARVERSTER demonstrated that the *K* value had the highest peak at *K* = 9, inferring that nine populations can incorporate all individuals from the 46 accessions with the highest likelihood. The structuring with *K* = 9 is shown in Fig. 3. The predicted population structure for the accessions displayed partial membership to more than one population, and none of the accessions showed membership to only one population. Several accessions, including the genetically most diverse accession (32713), had partial membership to eight different populations. However, in accessions with no or very low genetic variation, such as the two Norwegian accessions and accession 32048, the vast majority of their alleles came from a single population. Similar to cluster analysis, the distinctness of accession 32048 was depicted in the STRUCTURE analysis (Fig. 3) in that only very few other accessions share partial population membership (at very low proportion) with this accession. Furthermore, the structure analysis corroborates what is shown in the cluster analysis (Fig. 2a) in that there is no clear segregation of accessions based on geographic origin.

## Discussion

### EST-SSR Markers

New EST-SSRs for *P. sativum* subsp. *sativum* were developed from publicly available EST sequences in the present study. 73 % of these microsatellites are trinucleotide repeats whereas the remaining markers are either dinucleotide or hexanucleotide repeats. A high number of trinucleotide repeats among EST-SSRs is common in most cereal crops as well as in *P. sativum* [13, 27, 28]. Trinucleotide and hexanucleotide repeats are the most common SSRs, as length alterations of these SSRs rarely causes significant frame shift mutations during transcription [27, 29, 30]. Hence, such neutral length variations of SSRs are tolerated and passed to the next generation. About 40 % of the SSR loci were consistently amplified across the *P. satium* ssp. *sativum* accessions used during the development of these markers, which is similar to the work of Mishra et al. [13]. 80 % of these loci were polymorphic, which is slightly higher than that reported by Burstin et al. [27].

EST-SSR markers have an advantage of higher transferability to related genera as compared to SSRs from non-coding regions of a genome. This is due to the fact that they are located in the highly conserved transcribed region of the genome, with low vulnerability to random mutation [27, 31, 32]. In this study, 93 % of the SSR loci were shared by all taxa within the genus *Pisum*, and about 47 % of the loci were shared by all taxa studied. All loci except one were amplified in *P. fulvum* whereas less than 70 % of the loci were amplified in *V. faba* and *L. culinaris*. This is in line with the general trend that the across-taxa transferability of EST-SSR markers decreases with an increase in genetic distance between species [33]. Despite the successful amplification of most markers in the *Pisum* subspecies, the level of polymorphism was reduced as compared to that observed in *P. sativum* subsp. *sativum*. This reduced polymorphism in other *Pisum* subspecies could be attributed to the low number of individuals used as compared to that of *P. sativum* subsp. *sativum*.

### Genetic diversity

A total of 37 alleles were detected across 12 loci in 46 accessions of *P. sativum* subsp. *sativum*. PS10 was the most polymorphic locus with six alleles and a Shannon diversity index value of 0.96. Previous studies in *P. sativum* with EST-SSRs and genomic SSRs produced similar results as in this study with most polymorphic loci having seven alleles/locus [27, 34]. The average number of alleles per locus of 3.1 revealed in the present study is comparable with that of previous reports of 3.6 [27] and 3.8 [13] in SSR-based studies in *P. sativum*.

Despite the fact that all markers used for genetic diversity analysis were polymorphic, the observed heterozygosity was in general low for all loci with the highest being 0.05 for the locus PS13 (Table 3). The low heterozygosity is attributable to the fact that field pea is an inbreeder with cleistogamous flowers. The selfing nature of *P. sativum* could also be the reason behind the detection of private alleles in most of the loci studied. 25 % of the loci studied produced alleles unique to a single population (private alleles). Similarly, 25 % of the loci produced rare alleles restricted to one to two accessions with frequencies of less than 20 % and have overall frequencies of less than 1 % across the 46 accessions. In agreement with the present finding, Baranger et al. [35] reported rare alleles in *P. sativum* using EST-SSR loci. Rare and private alleles are of high implication as they might be in linkage disequilibrium with genes underlying desirable traits. Such alleles are important for tagging core collection sites as they could be exclusive to a specific population and/or locality [36]. It should be noted that mutations within a population will most likely remain within for selfing species like *P. sativum* and hence screening more accessions with these newly developed markers may lead to identification of more rare and private alleles of significance for field pea breeding.

The genetic diversity of most accessions is relatively high with more than 45 % of the accessions having Shannon diversity index of more than 0.3. Among the accessions studied, the most distinct was accession 32048. Unfortunately, the passport data for this accession was incomplete, and the only available information is that it was collected within Ethiopia. The present study revealed that this accession is distantly related to other accessions from Ethiopia; least diverse among the Ethiopian accessions and had several private alleles. The distinctness of this accession was clearly depicted in the cluster analysis (Fig. 2), as it was separated from the rest of Ethiopian accessions with 100 % bootstrap support. These data suggest that this accession may not be an Ethiopian landrace, but a variety introduced to Ethiopia from abroad. However, if indeed this accession is of Ethiopian origin, further investigations should be conducted to identify its unique traits, such as nutritional quality and disease and pest resistance. Among the three accessions included from NordGen, no genetic variation was detected within the two Norwegian accessions suggesting that they are pure lines.

Analysis of molecular variance (AMOVA) revealed significant differentiation among accessions, regional and altitudinal groups. The level of differentiation among altitudinal groups is much lower (2 %) than that revealed among regions within Ethiopia (8 %). Hence, regions should get priority over altitudes when planning germplasm collecting missions in new areas that have not been covered previously. Comparison of pooled accessions showed a significant difference with relatively low FST scores between the pooled groups. It is possible that the close relationship between accessions from different regions within Ethiopia could be a consequence of seed exchange among farmers resulting in low differentiation in allele distributions. However, the overall comparison revealed significant variation among accessions, P < 0.001 (Table 7), due to the presence of significant number of private and rare alleles as well as differences in allele frequencies among accessions.

The present study did not reveal clear pattern of clustering of accessions according to their geographic origin (Fig. 2). For example, accessions from USA and Norway were not clearly segregated from those originating in Ethiopia. Accessions from different region within Ethiopia were also clustered with no clear pattern. This is mainly attributable to gene flow between regions at different rates. Previous studies in *P. sativum* with morphological and molecular markers also showed the absence of correspondence between genetic distance and geographical distance [2, 6, 37, 38]. Mixed clustering pattern of accessions was highlighted in the STRUCUTRE software-based population structure (Fig. 3) in that the vast majority of the accessions had individual genotypes that show partial membership to multiple clusters. Structure simulation revealed that the highest *K* value is at L(*K*) = 9, inferring all 548 individuals can be grouped into nine populations with the highest probability (Additional file 4).

All Ethiopian accessions used in this study have been screened for resistance against pea weevil [5]. The mean percent seed damage (PSD) of these accessions during field trials conducted at three sites in Ethiopia ranged from 27 % (accessions 237945 and 32509) to 68 % (accession 32711), which is more than two-fold (see Table 5). No rare or private allele was recorded for the two accessions with the lowest PSD. However, accession 32730 (PSD = 29 %) has an allele that it shared only with accession 32048. Accession 32048, which is the most genetically distinct accession, had a PSD of 46 %. Such alleles might be linked to quantitative trait loci (QTL) conferring resistance against pea weevil in field peas and, if so, could potentially be used in marker assisted breeding to develop resistant varieties. Furthermore, most of the newly developed markers were successfully amplified in *P. fulvum*. Since *P. fulvum* is known to have enhanced resistance against pea weevil [39, 40], those markers that are polymorphic among the two species could be used for selecting interspecific hybrids between these two species in breeding program targeting the development of resistant varieties against this pest.

Given the fact that only five accessions were needed to capture all the 37 alleles identified in the present study (Table 4), the newly developed markers are highly suitable for the development of core collections of field peas. It is also important to note that no redundancy was observed among the EIB accessions, as each accession was unique in its overall genetic profile over the 12 loci studied. Similar to other studies on crops that are known to have long cultivation history in Ethiopia, such as niger [41, 42], barley [43] and sorghum [44], this study revealed high levels of genetic variation in field pea accessions from Ethiopia. The observed high genetic diversity implies the potential of the Ethiopian field pea gene pool for improvement of field peas in various desirable traits, including resistance to insect pests. These newly developed EST-SSR primer-pairs successfully amplified expected loci in *P. sativum* subsp. *sativum* as well as in other subspecies of the genus *Pisum* and related genera.

## Conclusions

In the present study, 15 new EST-SSR markers have been developed for *P. sativum* ssp. *sativum*. These markers were also successfully amplified in other *Pisum* taxa and other closely related species. Hence, these markers are highly valuable resources for various applications such as phylogenetic studies and genetic linkage mapping as well as for resolving the inconsistency in the taxonomic status of the different subspecies of the genus *Pisum*. Additionally, the markers are useful asset for assaying the large collections of field pea landraces in Ethiopia that are yet to be characterized, and contribute to the breeding and conservation strategies of *P. sativum*. This study revealed the presence of high genetic diversity within field gene pool in Ethiopia. The detection of several private alleles in this study suggests the significant population differentiation at expressed portion of field pea genome, which can be useful from breeding point of view.

## Additional files

Additional file 1:
**Passport data of** ***Pisum sativum*** **ssp.** *sativum* accessions used for primer development and genetic diversity analysis. (DOC 81 kb) Additional file 2:
**Species passport data used for across-taxa transferability test of newly developed microsatellite markers.** (DOC 37 kb) Additional file 3:
**Passport data of** ***Pisum sativum*** **ssp.** *sativum* L. varieties from NordGen that were used for genetic diversity analyses. (DOC 28 kb) Additional file 4:
**Graphical representation of mean likelihood L(K) and variance per K values generated by STRUCTURE on dataset containing 548 individuals across 12 polymorphic EST-SSR loci.** (JPEG 51 kb)

## Abbreviations

EIB: Ethiopian Institute of Biodiversity
EST: Expressed sequence tags
SSR: Simple sequence repeat
